# Application of the Electrical Microbial Growth Analyzer Method for Efficiently Quantifying Viable Bacteria in Ready-to-Eat Sea Cucumber Products

**DOI:** 10.3390/microorganisms12112301

**Published:** 2024-11-12

**Authors:** Xiaoyang Wang, Ruohan Liang, Xiaodan Pu, Yuanyuan Zhang, Feng Lu, Qianqian Yang, Xueting Zhu, Qing Kong, Xuzhi Zhang

**Affiliations:** 1 State Key Laboratory of Mariculture Biobreeding and Sustainable Goods, Yellow Sea Fisheries Research Institute, Chinese Academy of Fishery Sciences, Qingdao 266071, China; xyw3415@163.com (X.W.); a15238074419@163.com (R.L.); xiaodan_pu@163.com (X.P.); zyy15138589427@163.com (Y.Z.); lf200408@outlook.com (F.L.); qqyang_w@163.com (Q.Y.); z13188129281@163.com (X.Z.); 2 College of Food Science and Engineering, Ocean University of China, Qingdao 266003, China

**Keywords:** foodborne bacteria, quantitative assay, ready-to-eat seafood, culture-based method, growth curve

## Abstract

Accurate and efficient quantification of viable bacteria in ready-to-eat food products is crucial for food safety and public health. The rapid and accurate assessment of foodborne bacteria in complex food matrices remains a significant challenge. Herein a culture-based approach was established for easily quantifying viable bacteria in ready-to-eat sea cucumber (RSC) products. Samples of the liquid companion within the package were directly transferred into test tubes to determine bacterial growth curves and growth rate curves, utilizing the electrical microbial growth analyzer. Viable bacteria in the samples were then quantified based on the time required to attain the maximum growth rate indicated on the growth rate curve. At a concentration of 5.0 × 10^3^ CFU/mL of viable bacteria in the liquid companion, the recovery rates were 108.85–112.77% for *Escherichia coli* (*E. coli*) and 107.01–130.54% for *Staphylococcus aureus* (*S. aureus*), with standard deviations of 1.60 and 3.92, respectively. For the solid content in the package, the quantification was performed using the same methodology following an additional homogenization step. At a concentration of 5.0 × 10^3^ CFU/mL of viable bacteria in the sample, the recovery rates were 91.94–102.24% for *E. coli* and 81.43–104.46% for *S. aureus*, with standard deviations of 2.34 and 2.38, respectively. In instances where the viable bacterial concentration was 5.0 × 10^3^ CFU/mL in RSC products, the total time required for the quantification did not exceed 10.5 h. This method demonstrated advantages over traditional plate counting and PCR methods regarding simplicity and efficiency, representing a promising alternative for the quantification of viable bacteria in food like RSC products.

## 1. Introduction

Extensive research has demonstrated the presence of various bioactive compounds in sea cucumber, such as polypeptides, triterpene glycosides, glycoproteins, and sulfated fucan [1,2]. Due to its significant nutritional profile, sea cucumber has historically been esteemed as a vital medicinal and dietary resource in China and numerous other Asian nations [3]. In particular, ready-to-eat sea cucumber (RSC) has gained popularity because of its ease of consumption and the minimal degradation of nutrients and bioactive constituents [4]. However, both consumers and producers express considerable concern regarding the presence of viable microorganisms in RSC products. On one hand, viable microorganisms can lead to autolysis [3]; on the other hand, they may present substantial health hazards since these products are consumed without further cooking or processing [5]. Therefore, the ascertainment of viable microorganisms in RSC products is imperative throughout the stages of processing, distribution, and storage [6].

A range of methodologies has been developed for the detection of foodborne pathogens in food matrices [7]. Since the 19th century, the culture-based plate count technique has been employed to quantify bacterial populations [7,8]. To address its limitations, including labor intensity and long-term consumption [9], considerable emphasis has been placed on the advancement of culture-independent approaches. For achieving rapid bacterial detection, techniques such as microscopy, fluorescence-based assays, molecular diagnostics, immunological assays, and biosensors have been explored [10]. Microscopy allows for the direct and rapid enumeration of target bacteria in transparent mediums [11]. On a microscopic level, cellular images can be generated with a hyperspectral microscope. Theoretically, any pathogen can be detected with a spectral fingerprint using hyperspectral images once a reference library from pure bacterial isolates has been created. It can directly detect viable bacteria in complex food matrices [12]. Nevertheless, the necessity for observing individual cells, coupled with the demands for high magnification and a limited field of view, presents practical challenges in volume sampling, leading to potential inaccuracies in enumeration when applied to real samples [13]. Fluorescence-based techniques, such as flow cytometry, facilitate rapid measurements [14]. However, they encounter difficulties in distinguishing individual cells in non-fluid samples due to the heterogeneity, density, and similarity of cells and non-biological particles [15]. Raman spectroscopy, particularly surface-enhanced Raman spectroscopy, represents a promising modality for bacterial detection, owing to its exceptional sensitivity, real-time response, and capacity for molecular fingerprinting [16]. However, the robustness of the surface-enhanced Raman spectroscopy signal is dependent on the ability to concentrate the plasmatic particles in the area of the laser. Its accuracy would be interfered with by certain large particulates in food matrices [9]. In most cases, molecular-based methodologies such as PCR, qPCR, RT-PCR, DNA sequencing, and isothermal amplification typically bypass the need for culture, providing opportunities for the rapid detection of bacteria from complex matrices [17]. Nonetheless, these methods generally require sophisticated instrumentations, trained personnel, and intricate procedures involving DNA extraction, amplification, and identification [18]. Immunological methods often eliminate the need for a culture step and offer superior specificity compared to culture-based methods. However, strict control over antigen–antibody reaction conditions is crucial, and the complexity of sample preprocessing can sometimes lead to false positives [19]. Biosensors, including electrochemical variants, represent promising tools for the detection of bacteria in food [20,21,22]. However, traditional electrochemical techniques may face challenges such as electrode degradation and nonspecific binding. Moreover, invasive measurement approaches can yield inconsistent results that adversely affect accuracy [23].

Culture-based methods are still considered the gold standard up to now for the detection of foodborne pathogens [8], as they are superior to current non-culture-based methods in providing critical insights into the viability and metabolic activities of microorganisms, which are essential for understanding aspects such as metabolism, toxicity, autolysis, and spoilage. Assessing microbial viability and/or metabolic activity frequently necessitates prior sample treatments, such as the isolation and purification of target bacterial cells. These preparatory steps are prone to human error, often yield lower accuracy, and are labor-intensive [24]. Conceptually, the integration of automated, real-time monitoring of microbial growth within complex matrices can offer a viable approach for detecting foodborne pathogens in food products, via reducing the turnaround time and operation errors.

The capacitively coupled contactless conductivity detector (C^4^D) represents a specialized conductivity-based analytical technique wherein the electrodes remain insulated from the sample medium [11,25,26,27,28]. The intensity of the detected signal (C^4^ output) correlates directly with the concentration and mobility of the ionic charge carriers present in the medium. It retains the benefits of traditional contact electrochemical approaches, including simplicity of instrumentation, cost-effectiveness, rapid response, non-requirement for transparency, and ease of miniaturization. Moreover, it is free of polarization, passivation, and fouling risks [26]. It is well-known that microbial growth will transform uncharged or weakly charged substrates, such as yeast, peptone, and sugar, into highly charged end products, including amino acids and other metabolites, resulting in a conductivity increase in the medium [25]. Recently, our research group has advanced a novel concept for real-time monitoring of microbial growth by constructing a 32-channel electrical microbial growth analyzer (EMGA) based on developed C^4^D arrays [11,26]. Theoretically, the EMGA is capable of showing accurate and repeatable growth curves of well-dispersed and poorly dispersed microorganisms in real-time, regardless of whether they grow in homogeneous simple culture media or heterogeneous complex matrices. It has been utilized to evaluate the inhibitory effects of potassium sorbate and ZnO nanoparticles on *Escherichia coli* (*E. coli*) and *Staphylococcus aureus* (*S. aureus*) in milk-based beverages [29], as well as to investigate the antifouling performance of dendritic peptide-modified electrodes [30].

*E. coli* is considered to be one of the most dangerous pathogens responsible for the majority of food-borne outbreaks [31], while *S. aureus* was the causative agent in 2.87% of the Chinese foodborne outbreak [32]. According to Fang et al. [33], *E. coli* and *S. aureus* should not be detected in ready-to-eat foods. In this research, we established a straightforward culture-based approach for quantifying viable bacteria in RSC products through the utilization of the EMGA, using *E. coli* and *S. aureus* as model organisms. The characteristics of this novel methodology, such as its simplicity, efficiency, precision, and accuracy, were evaluated in comparison with classical plate counting and PCR methods.

## 2. Materials and Methods

### 2.1. Bacterial Strains

Standard bacterial strains of *E. coli* (ATCC35150) and *S. aureus* (ATCC25923) were purchased from BIOBW Biotechnology Co., Ltd. (Beijing, China) and were cultured according to a previously established protocol [11]. Briefly, strains were taken from −80 °C storage, seeded in Luria–Bertani (LB) broth, and (pre-grown) incubated in a shaking incubator (RADOBIO Scientific Co., Ltd., Shanghai, China) overnight at 37 °C. Subsequently, the active strains were transferred to fresh LB broth. Following an additional incubation period of approximately 10 h to reach the mid-exponential growth phase, the cell concentrations were determined using either the colony-forming unit (CFU) counting method or optical density measurement. The cultures were then promptly diluted to the required cell concentrations for further applications or centrifuged to pellet the cells for subsequent utilization.

### 2.2. Preparation of Artificially Contaminated Samples

RSC product samples were purchased from a local supermarket in Qingdao, China. Prior to the introduction of artificial contamination, the aseptic nature of these samples was verified using the LB agar plate method. The artificial contamination of the liquid companion sample was performed using a previously described protocol [11]. Briefly, *E. coli* or *S. aureus* cells were isolated from fresh LB broth by centrifugation (10,000 rpm for 15 min) and washed with 3× changes in water. The pelleted biomass was then re-suspended in water to constitute a bacterial suspension, whose concentration was determined by measuring the OD at 600 nm. Then, 10 µL of each bacterial suspension at known concentration was added to 1 mL of liquid companion, resulting in artificially contaminated samples with final bacterial concentrations of 0, 5.0 × 10^1^, 5.0 × 10^2^, 5.0 × 10^3^, 5.0 × 10^4^, 5.0 × 10^5^, and 5.0 × 10^6^ CFU/mL. Unless otherwise indicated, all samples were used within 2 h to prevent changes in viable cell counts. The artificial contamination of the solid content sample was carried out following the method reported by Jiang et al. [34] with minor modifications. Briefly, solid content and sterile saline (1:9 *w*/*w*) were homogenized using a Stomacher Lab-Blender (Masticator, IUL Instruments, Barcelona, Spain) at room temperature. Subsequently, 10 µL of the bacterial suspension was added to 1 mL of the homogenous mixture in a sterile centrifuge tube to generate artificially contaminated samples at final bacterial concentrations of 0, 5.0 × 10^1^, 5.0 × 10^2^, 5.0 × 10^3^, 5.0 × 10^4^, 5.0 × 10^5^, and 5.0 × 10^6^ CFU/mg.

### 2.3. Quantification of Bacteria in RSC Products with EMGA

Prior to quantifying bacteria in target RSC products with the EMGA method, working calibration curves with linear regression equations for *E. coli* and *S. aureus* in both liquid companion and solid content were established. Firstly, 1 mL of each liquid companion sample or homogenate of solid content artificially contaminated with bacteria at various concentrations was pipetted directly into a test tube (5 mm NMR tube, NORELL, Morganton, NC, USA), which had been preloaded with 1 mL of sterile LB broth. The test tubes were sealed with 0.22 μm/13 mm Millex syringe filters (Merck-Millipore, Darmstadt, Germany) to allow sterile gas exchange [26]. Subsequently, these tubes were inserted into the working channels of an ER832 EMGA (manufactured by eDAQ Pty Ltd., Sydney, Australia) to determine bacterial growth curves. Unless otherwise indicated, trials were performed in triplicate. The incubation temperature, sampling interval, excitation frequency, excitation level, reference channel, and sampling count were 37 °C, 60 s, 200 kHz, 90%, channel 1, and 1200, respectively. Following the determination of growth curves, a polynomial regression algorithm listed in the “Curve Fitting” function was used to smooth curves with a polynomial order of 15. The growth rate curves were then decomposed using the data analysis tool of the EMGA to ascertain the time required to attain the maximum growth rate (*T*_mgr_) for each growth curve [26]. The resultant *T*_mgr_ values were plotted against the logarithmic values of contaminated *E. coli* or *S. aureus* to generate the calibration curves.

**Figure 1:**
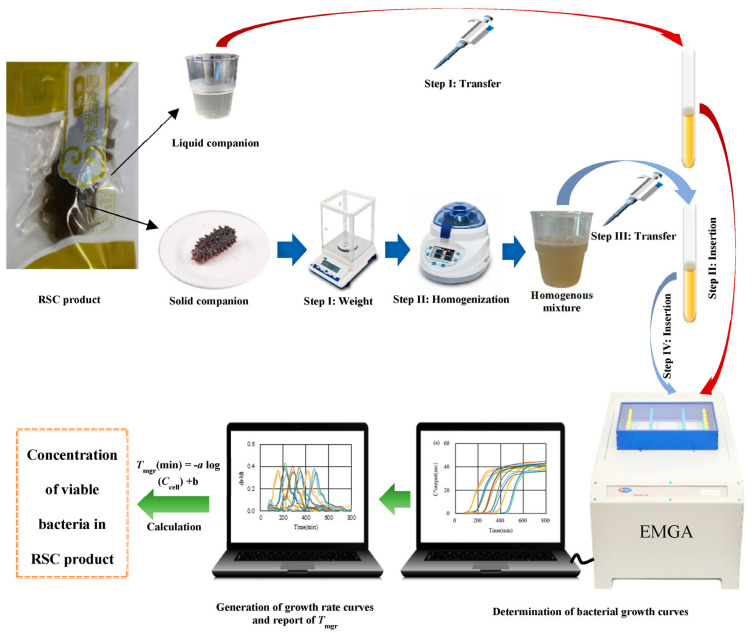
Schematic illustration for the quantification of viable bacteria in the liquid companion and solid content of RSC products with the EMGA method.

The quantification of concentration-unknown *E. coli* and *S. aureus* in the liquid companion of target RSC product(s) was executed with the procedure depicted in the schematic illustration (Figure 1). This process encompassed three primary stages: the transfer of samples to test tubes, the insertion of test tubes, and the determination of bacterial growth curves/growth rate curves with the EMGA. *T*_mgr_ values derived from the obtained growth rate curves were used to compute bacterial concentrations through established linear regression equations. For the quantification of viable bacteria in the solid content, the same protocol was applied, supplemented by two additional procedures: weight and homogenization.

### 2.4. Quantification of Bacteria in RSC Products with Plate Counting

A 25 mL aliquot of liquid companion, either contaminated with concentration-known *E. coli* and *S. aureus* or not, was extracted from the RSC product package. We generated 10-fold diluted culture solutions by mixing 50 μL of the liquid companion with 450 μL of physiological saline. Typically, six sequential 10-fold dilutions were created. For each dilution, three 100 μL aliquots were evenly distributed onto three LB agar plates (9.0 cm diameter). The plates were incubated overnight at 37 °C. The colony counts on the overnight plates, limited to 100 colonies, were recorded. The bacterial concentrations (CFU/mL) were calculated based on the average colony counts and the magnitude of culture dilution. Photographs of representative culture results were captured using a handheld Canon 90D camera.

A mass of 25 g of solid content was precisely measured and aseptically placed into a sterile stomacher bag, which had been pre-loaded with 225 mL of physiological saline. The specimen was then homogenized using a Stomacher Lab-Blender (Masicator, IUL Instruments, Barcelona, Spain). Following this, the homogenate, which contained either a known concentration of *E. coli* and *S. aureus* or was free of contamination, was diluted with a precise volume of physiological saline and allowed to incubate for 10 min at a temperature of 2–4 °C. Finally, the supernatant was collected for cultivation on LB agar plates.

### 2.5. Quantification of Bacteria in RSC Products with PCR

Contaminated liquid companion and homogenate of solid content were submitted to total genomic DNA extraction utilizing the TianGen (Tiangen Biotech (Beijing) Co., Ltd., Beijing, China) commercial total DNA extraction kit, according to the manufacturer’s instructions. The quality and concentration of the extracted total genomes were assessed using a Biodropsis BD-1000 spectrophotometer (Oriental Science & Technology Development Co., Ltd., Beijing, China). All extracted DNA was standardized to a concentration of 50 μg/mL prior to its application in real-time PCR assays. The detection of *E. coli* and *S. aureus* was performed utilizing the methods established by Pakbin et al. [35] and Yoon et al. [36], respectively (details are shown in Supplementary Materials).

### 2.6. Limit of Detection (LOD)

The LOD of the EMGA method was assessed using the strategy reported by Jiang et al. [34] with minor modifications. Briefly, the liquid companion and homogenate of the solid content were contaminated with *E. coli* or *S. aureus* at final concentrations of 5.0 × 10^0^, 5.0 × 10^1^, 5.0 × 10^2^, and 5.0 × 10^3^ CFU/mL. Subsequently, 1 mL of each contaminated sample was transferred to a test tube containing 1 mL of LB broth. The experiments were conducted in triplicate. Bacterial growth curves were determined with the EMGA. The presence of three distinct growth curves at the end of the 1200 min measurement indicated successful bacterial detection; conversely, a lack of growth curves or fewer than three curves suggested that the bacterial concentration fell below the LOD.

### 2.7. Recovery Rate

Liquid companion samples and homogenates of the solid content were artificially contaminated with *E. coli* or *S. aureus* at final concentrations of 5.0 × 10^1^, 5.0 × 10^2^, 5.0 × 10^3^, 5.0 × 10^4^, 5.0 × 10^5^, and 5.0 × 10^6^ CFU/mL, respectively. Subsequently, the concentrations were determined with the EMGA, plate counting, and PCR methods. All experiments were conducted in triplicate. The recovery rate was calculated as the percentage deviation of the experimental values from the nominal concentrations, expressed as recovery (%) = (measured value/theoretical value) × 100 [34].

### 2.8. Statistical Analysis

The recovery rates were expressed as the mean ± standard deviation (SD). Statistical and linear regression analyses were conducted using Microsoft Excel (version 2021), CellStatz (version 1.2), and Origin (version 2022). The evaluation of accuracy and precision was based on the recovery rate and its corresponding SD. Significance was set at the level of *p* < 0.05.

## 3. Results and Discussion

### 3.1. Bacterial Growth Curves and Growth Rate Curves

**Figure 2:**
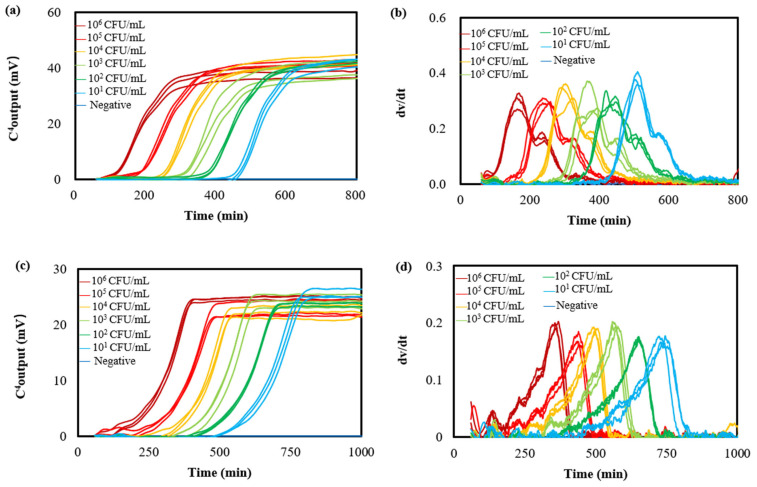
Triplicate growth curves (**a**) and growth rate curves (**b**) of *E. coli* determined with the EMGA. Triplicate growth curves (**c**) and growth rate curves (**d**) of *S. aureus* determined with the EMGA. *E. coli* and *S. aureus* were at varying concentrations in liquid companion samples.

Liquid companion samples were artificially inoculated with *E. coli* at concentrations of 0, 5.0 × 10^1^, 5.0 × 10^2^, 5.0 × 10^3^, 5.0 × 10^4^, 5.0 × 10^5^, and 5.0 × 10^6^ CFU/mL and distributed into test tubes in triplicate for each concentration. The bacterial growth curves were determined subsequently. Figure 2a shows typical results (details are shown in Supplementary Materials). Sigmoidal curves, which were similar to those obtained with conductivity [37], impedance [38], and optical density [39] methods, were present as expected, reflecting standard bacterial growth and proliferation. Utilizing the EMGA’s data analysis functionality, specifically the “first-order derivative” feature, the bacterial growth curves were decomposed into bell-shaped growth rate curves (Figure 2b). The peak values, including the maximum C^4^ output and *T*_mgr_, were automatically displayed in the graphic frame of these growth rate curves by clicking on the “first-order derivative” button. Serving as a key growth kinetic parameter, *T*_mgr_ offered an alternative definition for quantifying initial bacterial populations.

Liquid companion samples contaminated with *S. aureus* at final concentrations of 0, 5.0 × 10^1^, 5.0 × 10^2^, 5.0 × 10^3^, 5.0 × 10^4^, 5.0 × 10^5^, and 5.0 × 10^6^ CFU/mL were also evaluated. Figure 2c shows the resulting typical triplicate bacterial growth curves. The presence of sigmoidal curves indicates typical bacterial growth and proliferation, encompassing distinct phases of lag, acceleration, exponential growth, deceleration, and stationary periods. Notably, the growth curve profile of *S. aureus* exhibited a markedly different fingerprint compared to that of *E. coli*, implying divergent metabolic pathways. Furthermore, the growth rate curves (Figure 2d) indicate that the *T*_mgr_ for *S. aureus* is higher than that of *E. coli* at identical initial contamination levels, suggesting that *S. aureus* requires a longer duration to produce sufficient metabolic products to yield a detectable increase in conductivity. This finding corroborates our previous observation [11].

**Figure 3:**
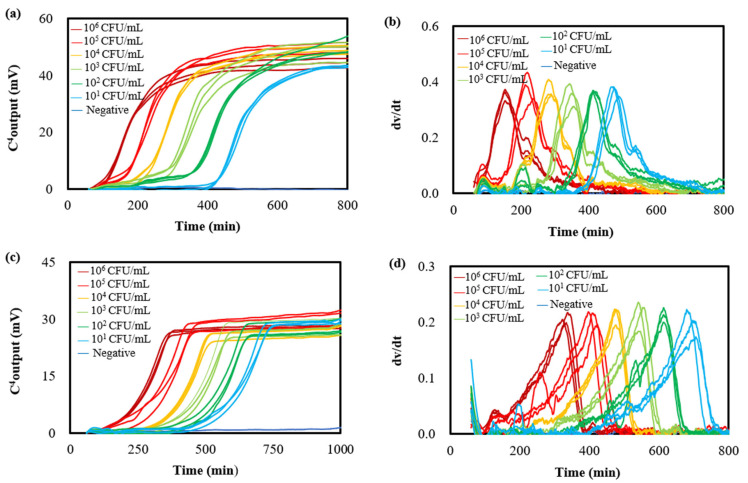
Triplicate growth curves (**a**) and growth rate curves (**b**) of *E. coli* determined with the EMGA. Triplicate growth curves (**c**) and growth rate curves (**d**) of *S. aureus* determined with the EMGA. *E. coli* and *S. aureus* were at varying concentrations in solid content samples.

Homogenate of solid content artificially contaminated with *E. coli* and *S. aureus* at final concentrations of 0, 5.0 × 10^1^, 5.0 × 10^2^, 5.0 × 10^3^, 5.0 × 10^4^, 5.0 × 10^5^, and 5.0 × 10^6^ CFU/mL were analyzed with the EMGA method. Resulting bacterial growth curves are shown in Figure 3a,b. The presence of the fragmented bio-tissues in these heterogeneous matrices exerted minimal influence on the shape of these bacterial growth curves. However, the presence of the fragmented bio-tissues increased the maximum C^4^ output values of *E. coli* and *S. aureus*, suggesting that the bacterial maximum growth was enhanced. This enhancement is likely attributable to the nutritional components (such as proteins, polypeptides, triterpene glycosides, glycoproteins, sulfated fucans, etc. [2]) derived from the solid content. Furthermore, when the contaminated *E. coli* and *S. aureus* were 10^6^ CFU/mL, the corresponding *T*_mgr_ values were 159 ± 2 min and 348 ± 2 min, respectively (as shown in Figure 3c,d). In contrast, when the contaminated *E. coli* and *S. aureus* were 10^6^ CFU/mL in liquid companion samples, the corresponding *T*_mgr_ values were 172 ± 3 min and 376 ± 2 min, respectively (as shown in Figure 2b,d). This indicates that the presence of comminuted solid content reduces the lag time of this bacterial growth.

### 3.2. Linear Range

**Figure 4:**
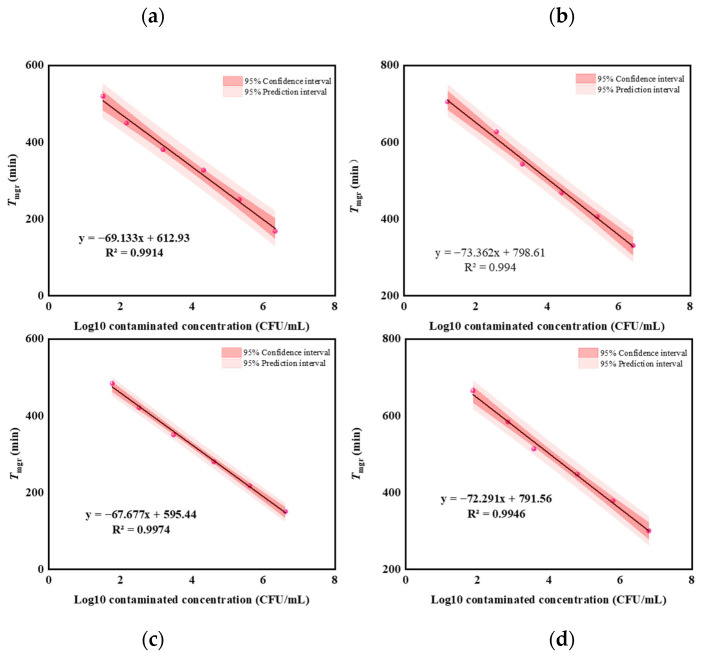
The linear relationships between the logarithmic concentrations of *E. coli* (**a**) and *S. aureus* (**b**) in liquid companion samples and the *T*_mgr_ values. The linear relationships between the logarithmic concentrations of *E. coli* (**c**) and *S. aureus* (**d**) in homogenates of solid content and the *T*_mgr_ values.

Figure 4a,b shows the relationships between logarithmic concentration values of *E. coli* and *S. aureus* in artificially contaminated liquid companion samples and the *T*_mgr_ values derived from Figure 2b,d. Over the concentration range of 5.0 × 10^1^ to 5.0 × 10^6^ CFU/mL, a direct proportionality was observed between the bacterial concentration and the *T*_mgr_, thereby facilitating the quantification of viable bacteria in liquid companions through a *T*_mgr_ determination via the EMGA method. The correlation coefficients of the regression lines surpassed those yielded by the impedance method for viable *E. coli* detection in urine samples (r > 0.9000) [31].

Figure 4c,d shows the relationships between logarithmic concentration values of *E. coli* and *S. aureus* in artificially contaminated homogenates of solid content and the *T*_mgr_ values derived from Figure 3b,d. Over the concentration range of 5.0 × 10^1^ to 5.0 × 10^6^ CFU/mL, a direct proportionality was observed between the bacterial concentration and the *T*_mgr_. The correlation coefficients of the regression lines demonstrated superiority over the indirect impedance method employed for assessing viable *L. innocua* in zucchini purée and béarnaise sauce matrices (r ≥ 0.82) [22].

### 3.3. Simplicity and Efficiency

Culture-based methods for detecting viable bacteria in food samples necessitate a series of steps, including dilution, transfer, spreading, enumeration, and computation, followed by parallel subculture [7]. Factors such as the uneven distribution and low prevalence of pathogens in food matrices, the inherent heterogeneity of food substrates, and the interference from indigenous microbiota can compromise the precision and accuracy of culture-based outcomes [40]. Herein, the quantification of viable microorganisms in the liquid companion of RSC products was streamlined into three steps: transferring liquid companion samples into test tubes, inserting the test tubes into the EMGA, and determining bacterial growth curves. Unlike electrochemical approaches utilizing contact electrodes, this method eliminated the need for ancillary processes (e.g., centrifugation [31] and modification of electrode [41]) for treating target complex samples or measurement instruments.

Commonly, the plate counting method necessitates serial 10-fold dilutions and manual spreading of samples on agar plates prior to incubation [34], while the PCR method needs the extraction and purification of DNA and the manual preparation of gene amplification mixtures [35]. Conventional optical pattern techniques often demanded sample pretreatments to achieve a requisite level of transparency [15]. In contrast, in the EMGA method, the aforementioned procedural steps were rendered unnecessary due to the absence of concerns regarding turbidity and color interference. This omission allowed for the avoidance of related auxiliary equipment and procedural complexities, thereby significantly streamlining the analytical process.

When there was 10^3^ CFU/mL of viable *E. coli* or *S. aureus* in the liquid companion, the total time from the unsealing of the RSC product package to the acquisition of quantitative results was limited to a maximum of 10.0 h. In comparison, the plate counting method necessitates a minimum of 24 h [42], while real-time PCR requires approximately 5 h [35,36]. Of note, the reliability of PCR methods partially hinges on the purity of the nucleic acid template and the adequacy of target molecule quantities. Given the intricate nature of food matrices, specific procedural steps are essential to mitigate the impact of potential inhibitory substances that could compromise PCR amplification. Additionally, enrichment steps are frequently required to enhance the sensitivity [43], prolonging the detection time of PCR significantly. Consequently, the EMGA method demonstrated a marked reduction in both labor and time requirements relative to plate counting and PCR methods. When the concentration of *E. coli* in the liquid companion was 10^6^ CFU/mL, the quantification process was completed in approximately 6.0 h, qualifying it as a rapid analytical method [44].

The implementation of the EMGA method for quantifying viable bacteria in the solid content of RSC products necessitated two supplementary procedures: mass measurement and homogenization. This led to an additional time investment of approximately 0.5 h. Even so, this method demonstrated greater simplicity and efficiency when contrasted with conventional plate counting and PCR methods.

### 3.4. Sensitivity

For the quantification of viable bacteria in food samples, the average LODs of lateral flow immunochromatographic assay, electrochemical methods, and PCR were 24 CFU/mL, 12 CFU/mL, and 6 CFU/mL, respectively [45]. When the EMGA method was used for quantifying *E. coli* and *S. aureus* in both liquid companion and solid content of RSC products, the LOD was 10 CFU/mL(mg). In contrast, the plate counting method yielded LODs of 60 CFU/mL and 70 CFU/mL for *E. coli* and *S. aureus*, respectively, in the liquid matrix, and 20 CFU/mL and 30 CFU/mL in the solid content. Our findings suggest that the PCR method exhibits superior sensitivity, achieving LODs below 10 CFU/mL(g) for both bacterial species in the liquid and solid matrices of RSC products. These results imply that this novel method demonstrates comparable sensitivity to prevalent electrochemical approaches [41,44].

### 3.5. Accuracy and Precision

**Figure 5:**
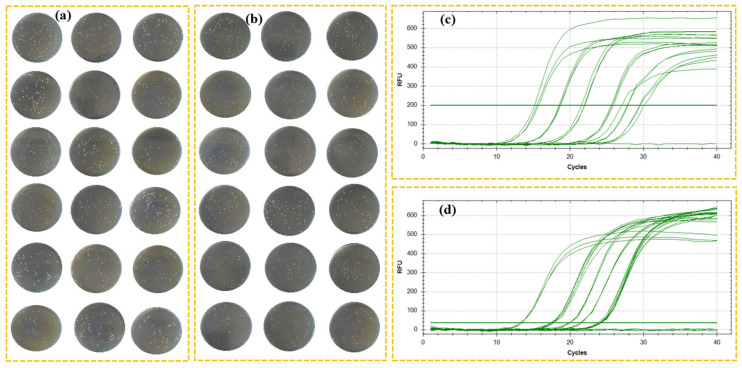
Typical LB agar plate images of *E. coli* (**a**) and *S. aureus* (**b**) at 5.0 × 10^1^, 5.0 × 10^2^, 5.0 × 10^3^, 5.0 × 10^4^, 5.0 × 10^5^, and 5.0 × 10^6^ CFU/mL (from top to bottom) in liquid companion samples. In case the initial concentration of bacteria in contaminated liquid companion samples was higher than 10^3^ CFU/mL, an appropriate dilution was used prior to the spreading step to make sure that the expected colony was below 100 on the agar plate. Typical PCR curves for template DNA from 5.0 × 10^1^, 5.0 × 10^2^, 5.0 × 10^3^, 5.0 × 10^4^, 5.0 × 10^5^, and 5.0 × 10^6^ CFU/mL (from right to left) of *E. coli* (**c**) and 5.0 × 10^1^, 5.0 × 10^2^, 5.0 × 10^3^, 5.0 × 10^4^, and 5.0 × 10^5^ CFU/mL (from right to left) of *S. aureus* (**d**).

Figure 5a,b show the typical agar images of *E. coli* and *S. aureus*, respectively, at the end of a 20 h incubation. The results clearly indicated a problem in repeatability, particularly at elevated bacterial concentrations. When liquid companion samples were contaminated with bacteria at high concentrations, they underwent more dilution steps. Each dilution introduced variability, compromising both repeatability and precision [46]. Figure 5c,d show the typical results of PCR amplification curves for template DNA from *E. coli* and *S. aureus*, respectively. As expected, there was a linear relationship between the *C*_t_ values and bacterial count [35], even though PCR was inadequate to discriminate between viable and non-viable bacteria.

Table 1 shows a summary of their recovery rates and SDs for these methods. For *E. coli*, the recovery rates obtained by the EMGA method ranged from 76.13 to 117.79%. In comparison, these rates were superior to those obtained by the plate counting method (74.28–123.78%) and PCR method (54.19–94.31%). For *S. aureus*, the recovery rates for the EMGA, plate counting, and PCR methods were 89.52–133.33%, 68.06–132.26%, and 51.71–97.65%, respectively. These results indicate that the EMGA method demonstrates superior accuracy in quantifying both *E. coli* and *S. aureus* in liquid companion samples. For both *E. coli* and *S. aureus*, the EMGA method and the plate counting method showed similar (*p* > 0.05) recovery rates, while substantial differences were observed between the EMGA method and the PCR method (*p* < 0.05). Furthermore, for quantifying *E. coli* and *S. aureus*, the SDs of the EMGA, plate counting and PCR methods were 1.60–9.95, 5.93–17.56, and 4.81–14.58, respectively, highlighting that the EMGA method offers the greatest precision.

Contaminated *E. coli* and *S. aureus* at various concentrations in homogenates of solid content were also quantified using the plate counting and PCR methods, respectively, in triplicate. Table 2 shows the summary of the recovery rates and SDs of these methods alongside the EMGA method. For *E. coli*, the EMGA method yielded recovery rates between 82.14% and 116.46%. In contrast, the plate counting method reported recovery rates ranging from 71.53% to 116.32%, consistent with the findings of Jiang et al. [34] (78.36% to 107.38%). The PCR method demonstrated recovery rates from 63.76% to 98.31%, aligning with the results of Fukushima et al. [47] (average of 56.8%). Regarding *S. aureus*, the recovery rates for the EMGA, plate counting, and PCR methods were 74.51% to 110.68%, 68.85% to 116.32%, and 62.42% to 97.59%, respectively. Our findings for the plate counting method corresponded with those (70.01% to 104.23%) of Jiang et al. [34], while the PCR method results were consistent with the data reported by Francois et al. (60% to 85%) [48]. For both *E. coli* and *S. aureus*, the EMGA method and the plate counting method showed similar (*p* > 0.05) recovery rates, while substantial differences were observed between the EMGA method and the PCR method (*p* < 0.05).

Several factors, including the manual handling during pretreatments, the intrinsic properties of the samples, and the substantial quantities of food components, can significantly affect the results of quantitative analysis. Bacterial concentration in solid samples obtained by the plate counting method was occasionally several orders of magnitude different from that determined with the PCR method [47]. Research indicates that the extraction of bacterial cells from food samples and DNA extraction from bacterial cells can compromise the efficiency and accuracy of the quantification [49], explaining that the PCR method had the lowest recovery rates. In the case of both *E. coli* and *S. aureus*, the recovery rates of the EMGA method were superior to those of the plate counting and PCR methods, suggesting that the proposed method offers the highest accuracy for quantifying viable bacteria in solid content samples. Furthermore, the SDs of the EMGA (2.34–5.96), plate counting (5.09–24.97), and PCR (2.72–9.19) methods indicate that the EMGA method demonstrates the greatest precision. Additionally, its precision surpasses those of microbiological survey-based method [34], electrochemical sensor [20] and impedance method [22].

### 3.6. Cost

Unlike conventional electrochemistry-based methods, chemical (e.g., pH-sensitive fluorescent nanoparticles [50]) and biotic (e.g., aptamer [51]) indicators/auxiliary materials are not needed. The reagents and consumables utilized in the EMGA method are limited to culture broth, sterile saline, test tubes, micropipette tips, centrifuge tubes, and a sterile homogenizer bag. Approximately USD 1 per sample is needed for the quantification of viable bacteria in RSC products. In contrast, the costs of the plate counting and PCR methods are approximately USD 2 and USD 8 per sample, respectively. Consequently, the quantification of viable bacteria in food matrices with the EMGA method proves to be economically advantageous concerning instrumentation, reagents, and consumables.

### 3.7. Limitations and Further Investigations

While *E. coli* and *S. aureus* are regarded as standard models and are often employed as comparative targets in biological research [11,34], actual RSC products may exhibit contamination by other bacterial species (e.g., *Vibrio parahaemolyticus*) or a combination of various bacteria. Consequently, future investigations will focus on assessing the specificity of the EMGA method. The development of targeted quantification methods may benefit from the utilization of selective culture media and bacteriophages. Furthermore, it was observed that the bacterial concentrations obtained with the EMGA method were consistently higher than those determined with the plate counting method, particularly when the bacteria were present in the solid content of RSC products. This discrepancy is intriguing and warrants further exploration to elucidate its underlying causes.

## 4. Conclusions

Utilizing the electrical microbial growth analyzer to determine bacterial growth curves, a culture-based method was established for quantifying viable bacteria in RSC products. This was the first introduction of this detection method to food safety. For the liquid companion within the packaging, samples were directly transferred into test tubes without pretreatment steps. For the solid content, assays were performed with the same procedures, following an additional homogenization step. The EMGA method eliminates costly and labor-intensive pretreatment steps, demonstrating superiority over traditional plate counting and PCR methods in terms of simplicity, efficiency, accuracy, precision, and cost-effectiveness. With further development of the method’s specificity in future studies, it will be a promising alternative for quantifying bacteria’s presence in a variety of food products.

## Figures and Tables

**Table 1:** Recovery rates and SDs of the EMGA, plate counting, and PCR methods for quantifying bacteria in liquid companion of RSC products.

Strains	Concentration CFU/mL	EMGA		Plate Counting		PCR	
		Recovery Rate (%)	SD	Recovery Rate (%)	SD	Recovery Rate (%)	SD
*E. coli*	5.0 × 10^6^	89.05–94.36	2.50	74.28–98.63	10.60	69.66–80.75	4.81
	5.0 × 10^5^	91.52–112.93	5.84	78.12–85.71	9.10	79.15–83.77	6.12
	5.0 × 10^4^	101.44–117.79	6.77	93.44–123.78	11.43	54.19–81.60	11.20
	5.0 × 10^3^	108.85–112.77	1.60	86.66–114.74	6.36	70.00–94.31	10.04
	5.0 × 10^2^	87.37–96.71	3.83	85.71–101.40	7.32	67.51–86.41	8.20
	5.0 × 10^1^	76.13–81.38	2.22	80.20–89.00	9.43	68.90–81.36	11.85
*S. aureus*	5.0 × 10^6^	89.52–110.99	5.86	68.06–87.72	12.11	63.29–93.09	12.71
	5.0 × 10^5^	103.04–125.81	5.25	96.00–132.26	15.64	88.12–92.33	4.82
	5.0 × 10^4^	96.88–123.75	3.76	86.49–113.51	12.40	83.06–97.65	6.27
	5.0 × 10^3^	107.01–130.54	3.92	88.24–102.70	5.93	51.71–86.66	14.27
	5.0 × 10^2^	95.20–117.28	8.19	78.26–108.33	13.18	60.84–83.31	14.58
	5.0 × 10^1^	104.28–133.33	9.95	50.00–114.29	17.56	Not available	Not available

**Table 2:** Recovery rates and SDs of the EMGA, plate counting, and PCR methods for quantifying bacteria in the solid content of RSC products.

Strains	Concentration CFU/mL	EMGA		Plate Counting		PCR	
		Recovery Rate (%)	SD	Recovery Rate (%)	SD	Recovery Rate (%)	SD
*E. coli*	5.0 × 10^6^	100.70–110.82	3.54	88.70–116.32	13.04	82.90–98.31	6.39
	5.0 × 10^5^	97.72–106.88	3.92	71.53–110.63	22.21	94.21–98.02	4.78
	5.0 × 10^4^	87.15–116.46	2.67	98.36–112.90	6.86	65.70–82.00	7.13
	5.0 × 10^3^	91.94–102.24	2.34	82.14–95.24	5.75	88.91–94.72	4.79
	5.0 × 10^2^	82.14–95.24	3.75	82.40–106.43	9.93	72.52–85.14	3.50
	5.0 × 10^1^	91.80–109.68	5.91	89.78–108.59	24.97	63.76–80.98	7.14
*S. aureus*	5.0 × 10^6^	103.92–110.68	2.96	71.78–85.00	8.67	62.42–92.37	9.19
	5.0 × 10^5^	95.39–109.73	3.62	90.28–116.32	6.25	87.81–95.30	4.64
	5.0 × 10^4^	94.51–108.70	2.84	69.57–70.42	9.26	83.67–97.59	5.04
	5.0 × 10^3^	81.43–104.46	2.38	73.28–71.43	5.09	89.82–93.56	2.72
	5.0 × 10^2^	76.99–89.48	4.10	75.96–106.99	14.21	69.63–85.69	5.63
	5.0 × 10^1^	74.51–83.06	5.96	68.85–73.68	19.80	Not aviable	Not aviable

## Data Availability

The data presented in this study are available on request from the corresponding authors.

## Supplementary Materials

The following supporting information can be downloaded at: https://www.mdpi.com/article/10.3390/microorganisms12112301/s1.
