# Supplementary material for: Application of the Electrical Microbial Growth Analyzer Method for Efficiently Quantifying Viable Bacteria in Ready-to-Eat Sea Cucumber Products
Source: Microorganisms. 2024 Nov 12;12(11):2301. doi: 10.3390/microorganisms12112301 (PMC11596173; doi:10.3390/microorganisms12112301)
Supplement: Supplementary file 1 [file microorganisms-12-02301-s001.zip › microorganisms-3277650-supplementary.pdf]

Supporting Information for

Application of the Electrical Microbial Growth Analyzer Method for Efficiently Quantifying Viable Bacteria in Ready-to-Eat Sea Cucumber Products

Xiaoyang Wang[1,2], Ruohan Liang[1], Xiaodan Pu[1], Yuanyuan Zhang[1], Feng Lu[1], Qianqian Yang[1], Xueting Zhu[1], Qing Kong[2*], Xuzhi Zhang[1*]

[1] *State Key Laboratory of Mariculture Biobreeding and Sustainable Goods, Yellow Sea Fisheries Research Institute, Chinese Academy of Fishery Sciences, Qingdao 266071, China*

[2] *College of Food Science and Engineering, Ocean University of China, Qingdao, 266003, China*

Corresponding authors: E-mail: zhangxz@ysfri.ac.cn (X. Zhang);

E-mail: kongqing@ouc.edu.cn (Q. Kong)

Quantification of bacteria in RSC products with PCR

The detection of *E. coli* was performed utilizing the methodology established by Pakbin et al., [1], employing a TB Green (TAKARA) on a CFX 96 Touch Real-Time PCR (Bio-Rad). Serogroup-specific primers, the forward (5'-CGGACATCCATGTGATATGG-3') and the reverse (5'-TTGCCTATGTACAGCTAATCC-3'), targeting *the rfbA* gene with primers were used. Each real-time PCR reaction contained 5 μL of the TB Green, 0.2 μL of each primer (20 μM), 1 μL of the DNA template and 4.8 μL of sterilized nuclease-free water. Amplification conditions were: initial denaturation step at 94°C for 5 min, followed by 40 cycles of denaturation at 94°C for 40 s, annealing at 60°C for 40 s and extension at 72°C for 30 s, followed by a melting step with the increasing temperature from 70 to 95°C with the raising temperature rate of 0.2°C/s. Each run included a negative control using distilled water and a positive control consisting of 10 ng/μL of pure DNA isolated from *E. coli*. A standard curve was constructed based on 10-fold serial dilutions of DNA derived from an *E. coli* cell suspension, with DNA quantities reflecting concentrations from 5.0×10^1 to 5.0×10^6 CFU/mL, amplified in triplicate via real-time PCR. For samples with unknown concentrations, the corresponding cell counts were determined by comparing the C_t values against the established standard curve [2].

The detection of *S. aureus* was executed utilizing the protocol established by Yoon et al. [2]. Specific forward (5′-CAT CGG AAA CAT TGT GTT CTG TAT G-3′) and reverse (5′-TTT GGC TGG AAA ATA TAA CTC TCG TA-3′) primers were used to amplify a targeted 94 base pair Sa 442 DNA fragment. Amplification conditions were: an initial denaturation step at 95°C for 10 minutes and 40 cycles at 95°C for 15 s and 60°C for 1 min. To confirm the specificity of the amplified products, a melting curve analysis was performed by gradually increasing the temperature from 60°C to 95°C at a rate of 0.3°C per cycle. Each run included a negative control of distilled water and positive control of 10 μg/mL pure DNA extracted from *S. aureus*.

Bacterial growth curves and growth rate curves

Liquid companion samples were artificially inoculated with *E. coli* at concentrations of 0, 5.0×10^1, 5.0×10^2, 5.0×10^3, 5.0×10^4, 5.0×10^5 and 5.0×10^6 CFU/mL and distributed into test tubes in triplicate for each concentration. The bacterial growth curves were determined subsequently. On the obtained sigmoidal curves, lag phases were evident, likely attributable to the stress induced in the bacteria during the dilution and loading processes, as well as the duration required to accumulate sufficient metabolic byproducts to elicit a detectable rise in conductivity [3]. The lag phase was succeeded by an acceleration phase, where the growth rate increased sharply, leading to the exponential phase. During the exponential phase, the growth rate slopes remained consistent across all samples, regardless of the initial *E. coli* concentrations. Following this, the growth rate began to diminish, entering a deceleration phase. Ultimately, the C^4 output values converged at similar levels, indicating that for *E. coli*, an increase in inoculum size expedited the observable initiation of growth, rather than enhancing the maximum growth rate or yield. In contrast, the negative control samples (devoid of *E. coli* contamination) displayed horizontal lines instead of sigmoidal growth curves throughout the incubation period, signifying that the absence of viable bacteria resulted in no variation in conductivity during the incubation and measurement.

Reference

[1] Pakbin, B., Brück, WM., Brück, TB., Allahyari, S., Tamai, IA. (**2023**). A quantitative prevalence of *Escherichia coli* O157 in different food samples using real-time qPCR method. *Food Sci Nutr* 11:228-235.

[2] Yoon, J-H., Wei, S., Oh, D-H. (**2018**). A highly selective enrichment broth combined with real-time PCR for detection of *Staphylococcus aureus* in food samples. *Lwt*, 94:103-110.

[3] Zhang, X., Yang, Q., Ma, L., Zhang, D., Lin, W., Schlensky, N., Cheng, H., Zheng, Y., Luo, X., Ding,

C., Zhang, Y., Hou, X., Lu, F., Yan, H., Wang, R., Li, C., Qu, K. (**2023**). Automatically showing microbial growth kinetics with a high-performance microbial growth analyzer. *Biosens Bioelectron*, 239:115626.
